# Assessment of the Bacterial communities associated with *Anopheles gambiae* larval habitats in Southern Ghana

**DOI:** 10.1371/journal.pone.0323464

**Published:** 2025-05-27

**Authors:** Akua Obeng Forson, Isaac Kwame Sraku, Idan Baah Banson, Jones Gyamfi, Kwabena Obeng Duedu, Yaw Asare Afrane

**Affiliations:** 1 Centre for Vector-borne Diseases Research, Department of Medical Microbiology, University of Ghana Medical School, University of Ghana, Korle-Bu, Accra, Ghana; 2 Department of Medical Laboratory Science, School of Biomedical and Allied Health Sciences, University of Ghana, Korle-Bu, Accra, Ghana; 3 Department of Biomedical Sciences, University of Health and Allied Sciences, Hohoe, Volta Region, Ghana; Guangzhou University, CHINA

## Abstract

Mosquito breeding habitats are ecosystems that comprise a complex, intimately associated micro-organism. This study aimed to determine the bacteria communities associated with *Anopheles* larval habitats and correlate their prevalence to the absence or presence of mosquito larvae. The 16S rRNA profiles of bacterial communities in *Anopheles*-positive breeding habitats (productive and semi-productive habitats) and negative habitats (non-productive) from Southern Ghana were analyzed using the Oxford Nanopore’s MinION platform with water and larval samples. A total of 15 bacterial taxa were identified across all habitats based on productivity. Significantly, mosquito-positive breeding habitats (productive and semi-productive) had more bacterial diversity compared to mosquito-negative habitats (non-productive). Comparison of the composition of bacteria in the different habitat types revealed that non-productive habitats had a higher prevalence of *Epsilonproteobacteria* (58.1%), while *Gammaproteobacteria* (33.2%) and *Betaproteobacteria* (30.5%) were dominant in the productive and semi-productive habitats. *Gammaproteobacteria* and *Betaproteobacteria* were the most abundant bacterial classes in *Anopheles* larvae. Comparing the water samples to larvae microbiomes revealed distinct composition. *Betaproteobacteri*a (58.5%) and *Cytophagia* (10.7%) were predominately present in the water samples, whilst *Betaproteobacteria* (47.9%) and *Gammaproteobacteria* (21.6%) were dominant in the larval samples. This study revealed a higher bacterial composition may play a role in *Anopheles* mosquitoes’ attractiveness to a breeding habitat. These findings contribute to the understanding of which bacteria, directly or indirectly, can be linked to the absence or presence of mosquito larvae in breeding habitats and set the basis for the identification of specific bacterial taxa that could be harnessed for vector control in the future.

## Introduction

The dependency on insecticides in the last decades has led to widespread resistance to major classes of insecticides used in vector control in sub-Saharan Africa and this threatens the control of vector-borne diseases, most especially malaria [1,2]. Novel vector control strategies are required to complement existing vector control strategies [3]. The different microbial flora associated with mosquitoes have become promising avenues for the development of novel control strategies for vector-borne disease [4,5].

Mosquito larvae feed and live in a complex ecosystem in mutualistic relationships with both detritus and microorganisms that include bacteria, fungi, viruses, and protists [5]. Larval interactions with microorganisms in mosquito breeding habitats can influence larval development and adults’ fitness [6]. The resident microbiota, in particular, bacteria in mosquito breeding habitats, serve as food for the larvae, play a vital role in mosquito physiology [7,8], and aid in attracting and repelling ovipositing female mosquitoes [9–14]. Studies on symbiotic microbiota in mosquitoes acquired from the breeding habitats have revealed they can play key roles in mosquito immunity [15,16], disease transmission [4,17,18], and host metabolism [8,19], making them valuable potential tools to combat mosquito-borne diseases.

In Ghana, malaria is a major public health problem and was responsible for an estimated 5.5 million illnesses and 308 deaths in 2022 [20]. The primary malaria vectors in Ghana are the *Anopheles gambiae* sensu lato (*An. gambiae* s. s*., An. arabiensis, An. coluzzii* and *An. melas*) and *Anopheles funestus* s. s*.* [21–24]*.* However, in view of the increasing concern about insecticide resistance hampering vector control strategies and the resurgence of malaria transmission in Africa [25,26], there is a need to explore novel intervention strategies for controlling its transmission by having a better understanding of the microbiota associated with *Anopheles* mosquitoes and their breeding sites using next-generation sequencing [27]. This is crucial for understanding which microbes are directly or indirectly related to the presence or absence of mosquito larvae in a breeding habitat and will provide a guide for developing innovative integrated vector control approaches in the future.

Some studies have investigated mosquito breeding sites for bacterial community composition and correlated this to the presence or absence of mosquito larvae. In New Orleans, Ponnusamy *et al*., [10] study reported the mean bacterial populations in water samples containing *Aedes* mosquitoes were found to be independent of container types and spatial distribution of containers. In Thailand, Dada *et al*., [7] evaluated the bacterial communities associated with *Ae. aegypti* larvae, and water from domestic water containers revealed no significant difference in operational taxonomic units’ abundance between mosquito-positive and negative water samples. Nilsson *et al*., [28] study with breeding sites in Brazil for *Anopheles darlingi* revealed the habitats were dominated by *Proteobacteria* and *Firmicutes*. Although studies conducted in Kenya and Cameroon by Onchuru *et al*., [29] and Gimonneau *et al*., [30] have reported that the predominant microbiota in the larval habitats of *An. coluzzii* and *An. gambiae* are *Firmicutes*, *Actinobacteria*, *Proteobacteria*, and *Bacteroidetes*, limited information is available on the microorganisms associated with the breeding waters of the dominant malaria vectors in Ghana. This study aimed to characterize the bacterial community composition in the *Anopheles gambiae* larvae and breeding habitats and correlate their prevalence to the absence or presence of mosquito larvae. We did this to assess whether there were correlations between the bacterial community composition and the *Anopheles* mosquito larvae they contained. This study has made it possible to analyze bacterial communities and identify particular bacterial populations that proliferate in habitats that are either productive (i.e., mosquito larvae are present) or non-productive (i.e., mosquito larvae are absent).

## Materials and methods

### Study sites

Samples of surface microlayer water and larval samples were collected from two sites in Southern Ghana: Anyakpor (5°46′51.96″N, 0°35′12.84″E) in the Ada East District and Dodowa (5° 52’ 58.3212’‘ N 0° 5’ 52.9548’‘ W) . The vegetation in Anyakpor is similar to that of the coastal savannah, and a majority of the population grows vegetables on raised beds with irrigated fields with irrigation systems that could serve as *Anopheles* mosquito breeding grounds.

However, Dodowa has a secondary forest type vegetation with little original virgin forest left as a result of deforestation that has created potential breeding habitats for mosquitoes. A total number of 59 mosquito larvae breeding habitats were identified within the study sites, with 18 in Dodowa (the savannah-forest transition area) and 42 in Ada (the coastal savannah area). These habitats were mapped using a Global Positioning System (GPS) and monitored over the 24 weeks (from October, 2020 to May, 2021) to record the stability and productivity. The habitats were surveyed once every two weeks for the presence of aquatic stages of *Anopheline* and to generate stage-specific estimates of larval densities as described previously [31].

### Mosquito larvae density estimation

At the time of collection of environmental samples, the density of *Anopheles* larvae was measured. Larvae were collected by standard dipping technique, using three dips with a 300 mL dipper of 9 cm diameter. The number of larvae was counted, and representative larvae were brought to the laboratory. In this study, we define a productive and non-productive habitat as a habitat with persistent presence and absence of anopheline larvae, and semi-productive habitat as habitats that switched between productive and non-productive habitats in no order at the time of habitat visitation and monitoring [31].

### Collection of larvae and water samples

From each productivity habitat type (productive, semi productive, and non-productive), water samples were collected directly into sterile-capped disposable centrifuge tubes. Water was vigorously mixed prior to sampling to ensure that biofilms were included. In addition, representative sample of 1^st^, 2^nd^, 3^rd^, and 4^th^ instar larvae (or all if less than ten) was collected from each mosquito-positive breeding habitat and transferred to sterile 15 ml Eppendorf tubes. All the samples were kept on ice and brought to the Medical Microbiology laboratory at the University of Ghana within 4 h of collection.

### Processing of larval and water samples

In the laboratory, each water sample was filtered through a cellulose nitrate membrane filter (0.22 μm pore size, 47 mm dia., Sartorius Stedium®) using aseptic vacuum filter units (Millipore®). Larval samples were surface sterilized; first rinsed in 70% ethanol, then suspended in 90% ethanol and agitated with a vortex mixer for about 10 seconds, and finally rinsed with sterile, DNA-free water. Immediately after filtration and larval surface sterilization, the samples were stored at -80^◦^C.

### Genomic DNA extraction

Samples were thawed at room temperature before DNA extraction, and each membrane filter was cut into small pieces and placed into sterile 1.5 ml Eppendorf tubes for DNA extraction. Bacterial genomic DNA was extracted from sterilized *Anopheles* larvae and bacterial cells retained on each filter membrane using the ZR Fungal/Bacterial/Algal DNA MicroPrep™ kit following the manufacturer’s instructions. Bacterial DNA extracted from the larvae and water samples were quantified with Nanodrop One™ (Thermo Fisher Scientific, USA) and stored at -20^o^C for subsequent PCR.

### PCR amplification

The full length of the 16S rRNA gene was amplified using the universal primers 27F 5’- AGAGTTTGATCCTGGCTCAG-3’ and 1492R 5’-GGTTACCTTGTTACGACTT-3’ [32,33], which yielded PCR fragments of approximately 1,500 bp in size. The PCR was set up with the NEB OneTaq 2X master mix (New England Biolabs, USA), 0.2 μM of each primer and 3 μl DNA template. Reaction conditions were as follows; 94˚C for 10 mins followed by 35 cycles of 94˚C for 30 secs., 58˚C for 1 min., 68˚C for 1min 30 sec. and a final extension at 68˚C for 5 mins. The amplification was done in triplicate and pooled for each sample. PCR products for each sample were examined on an agarose gel and cleaned using the QIAquick® PCR Purification Kit (QIAGEN GmbH, GERMANY). Purified PCR products were checked for purity using the NanoDropOne spectrophotometer (ThermoFisher Scientific, USA) and quantified with the Qubit dsDNA HS Assay Kit and read on the Qubit 4 Fluorometer (ThermoFisher Scientific, USA).

### Sequencing library preparation and sequencing

The sequencing library was prepared using the Native Barcoding Expansion 96 (EXP-NBD196) with the Ligation Sequencing Kit (SQK-LSK109) (Oxford Nanopore Technologies, UK) following the manufacturer’s instructions. Prepared libraries were pooled in equimolar amounts into a single library. The pooled library was sequenced using the MinION Mk1C device (Oxford Nanopore Technologies, UK) and a MinION R9.4.1 SpotON flow cell device according to the manufacturer’s instructions. Each run was set up for 72 hours.

### Sequence data processing

Sequencing reads (.fast5) were basecalled, demultiplexed, and trimmed of adapters and barcodes to.fastq using Guppy (Oxford Nanopore Technologies, UK). Quality control of the sequenced data was carried out with FastQC (https://www.bioinformatics.babraham.ac.uk/projects/fastqc/) through the Galaxy server. A quality score of 7 was given for each barcoded read. The minimum length filter was set at 200, and the maximum length filter was set at 1500. Minimum coverage was increased from the default level of 30× to 50×. Other parameters that were set included a minimum identity percentage of 80% and a maximum target sequence of 3.

### Data analysis

The taxonomy classification was performed g in Galaxy using Kraken2 against the PlusPF database (version: 2022-06-07) with a confidence threshold of 0.7 and a minimum hit group of 2. Output was clustered into operational taxonomic units (ASVs) at kingdom, phylum, class, order, family, and genus levels. The generated Kraken2 report file was used with sample metadata to generate the biological observation matrix (BIOM) file. All downstream analyses were performed in R (RStudio 203.12.0 build 369), using the phyloseq package. Visualization of bacterial composition abundances among samples, sampling sites, and larvae stages was done using bar charts showing the distribution of bacterial classes. Bar charts were created using the “ggplot2” package, and where required, a Venn diagram was generated using the “ggvenn” package in R. Venn diagrams were used to visualize shared ASVs when comparing between samples, sampling locations, habitats, and larvae stages. Alpha diversity was estimated using the observed species richness, Shannon diversity index (H) and chao richness. At various levels we compared the alpha diversity indices between groups with a one-way analysis of variance (ANOVA) followed by the Kruskal-Wallis test (followed by Dunn’s post hoc test if a significance difference were detected). Alpha diversity plots and statistical analysis were performed in R. Beta diversity between samples was estimated using the Bray-Curtis distance matrix method. Visualization for overall differences between bacterial communities’ structure between habitats types (Non-productive, Productive, and Semi-productive) and larvae stages (stage 1, stage 2, stage 3 and stage 4) was performed using nonmetric multidimensional scaling (NMDS). Permutational multivariate analysis of variance (PERMANOVA) and an analysis of similarity (ANOSIM) were used to determine significant differences between subgroups.

## Results

Approximately 6.3 million sequence reads were obtained from all 28 samples (S1 Table). A total of 5,070,324 (94.6%, Q-score = 11.71) sequences were used for further analyses after removing sequences shorter than 200 base pairs and potential chimera sequences. In the total of 28 samples, 5 samples were water filtrates from non-productive habitats; 4 samples from Ada-Foah and 1 sample from Dodowa, whereas 16 samples represented water samples from productive habitat samples; 10 samples being obtained from Ada-Foah and 6 from Dodowa. Based on this, 7 samples were classified as semi-productive due to their stability and productivity falling between that of productive and non-productive habitat samples. All 7 samples were obtained from Ada-Foah (S1 Table).

### Bacterial profiles and diversity across different sample types

**Fig 1:**
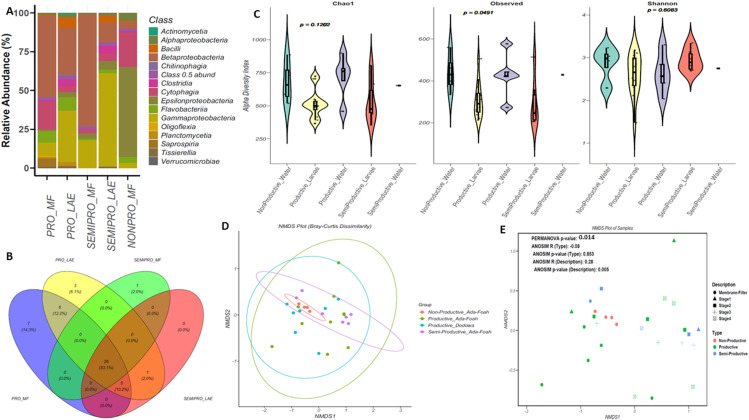
Bacterial profiles and diversity across different sample types. Bacterial community composition in An. gambiae larvae and water collected from breeding sites (Ada Foah and Dodowa) classified based on productivity of the habitats (Productive, semi-productive and non-productive) at the class taxonomy level. Only classes making up > 0.5% in any group of samples were included. Other classes present are clustered as “Class 0.5 abend” together with unknown classes (**B**) Mean alpha diversity metrics calculated within the various sample groups by (i) Observed species richness, (ii) Chao1, (iii) Shannon diversity index, (**C**) Four-way Venn diagram depicting ASVs numbers that overlap and do not overlap between various breeding habitat groups, (**D**) NMDS ordination plot based on Bray-Curtis distances and (**E**) NMDS ordination plot based on ANOSIM test. Each sample type is indicated by a different colour.

To compare samples across habitats reads from samples collected from water or larvae from specific habitats were merged into 5 specific sample types (Productive MF [PRO_MF], Productive Larvae [PRO_LAE], Semi-Productive water [SEMIPRO_MF], Semi-Productive larvae [SEMIPRO_LAE], and Non-Productive water [NONPRO_MF]). Using a 0.5% cut-off of relative abundance, a total of 15 taxa were identified at the class level across all habitats and larvae. The most abundant classes across the habitats were *Gammaproteobacteria, Betaproteobacteria* and *Epsilonproteobacteria*. For the non-productive habitat, the dominant class was *Epsilonproteobacteria* (58.1%), followed by *Cytophagia* (22.4%), the least occurring classes were *Bacilli* (0.65%) and *Clostridia* (0.63%) (Fig 1A). In the productive larvae, the predominant taxa were *Gammaproteobacteria* (33.2%) and *Betaproteobacteria* (30.5%) while the lowest taxa were *Chitinophagia* (0.81%) and *Actinomycetia* (0.64%). In the productive water habitat *Betaproteobacteria* dominated with 52.4% and was followed by *Cytophagia* with 18.6% (Fig 1A). The least occurring taxa in this habitat was the *Oligoflexia* (0.57%) and *Baccili* (0.55%). In the semi-productive larvae, the predominant taxa were *Gammaproteobacteria* (60.3%) and *Betaproteobacteria* (12.75%), while the lowest taxa were *Alphaproteobacteria* (1.48%) and *Tissierella* (0.95%). In the semi-productive water habitat, the predominant taxa were *Betaproteobacteria* (72.7%) and *Gammaproteobacteria* (18.0%) while the lowest taxa were *Flavobacteria* (0.86%) and *Alphaproteobacteria* (0.51%) (Fig 1A).

Each habitat type was characterised by unique bacterial composition. The most dominant class in the non-productive habitat was the *Epsilonproteobacteria* but was present in lower amounts in the productive and semi-productive habitats (Fig 1A). Interestingly, the bacterial composition in the water habitat mirrored what occurred in the *Anopheles* larvae. In the productive water and larvae, the *Betaproteobacteria* was a dominant taxon while in the semi-productive water and habitat *Gammaproteobacteria* was the dominant taxa*. Cytophagia, Clostridia,* and *Bacilli* were the classes identified between the productive and semi-productive habitats (Fig 1A). The *Actinomtcetia* was identified exclusively in the productive larvae, while *Tissierellia* was present in both productive and semi-productive larvae (Fig 1A). Furthermore, the most diverse bacteria was found in the productive samples, whereas fewer bacteria were found in the non-productive samples (Fig 1A, S1-S2 Figs).

Alpha diversity was estimated to determine the richness and evenness within the different habitat types (Fig 1B). Using the observed taxa (richness) measure at the class level, a statistically significant difference in taxa was observed (p = 0.0491, S2 Table). Water samples from the non-productive habitat had the highest mean observed diversity (959), followed by the water (919) and larvae (950) samples from the productive habitat. The least observed class taxa were the water sample (353) from the semi-productive habitat (Fig 1B [Observed]). Shannon and Chao1 alpha diversity indexes were not statistically significant (p = 0.6083 and p = 0.1202 respectively) (Fig 1B). Using the Shannon’s diversity index as measure for both richness and evenness, the water from the productive habitat had the highest mean index of 3.28, followed by the larvae samples from the productive habitat (3.15) (Fig 1B) [Shannon]).

Using a four-way Venn diagram, we compared the number of amplicon sequence variants (ASVs) that overlap across the habitats. The analyses revealed that 23 ASVs (53.1%) were shared between all the habitats (Fig 1C). Among these common taxa are *Actimomycetia, Alphaproteobacteria, Gammaproteobacteria, Clostridia* and *Deinococci*. The productive water habitat had the highest number of unique ASVs (7 [14.3%]), and 6 ASVs (12.2%) were shared exclusively between the productive water and larvae samples (Fig 1C, S3 Table). The non-productive water and the semi-productive larvae samples did not have any exclusive taxa. Four-way Venn diagram depicting ASVs that overlap and do not overlap between various breeding habitats at the genus and species taxonomic level is presented in S3 Table.

**Fig 2:**
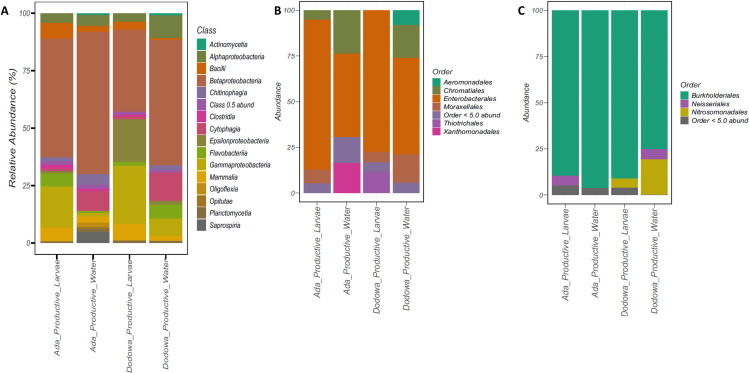
Bacterial community composition in *An. gambiae* breeding habitats and larvae in Ada Foah and Dodowa. Bacterial composition in An. gambiae water and larval samples collected from different breeding sites (Ada Foah and Dodowa) classified based on productive (Productive, semi-productive and non-productive) at class level (**B**) Dominant bacterial community composition at family level for Gammaproteobacteria. Only family making up > 0.1% in any sample are named, other family present are clustered as “Other” together with unknown the family (**C**) Dominant bacterial community composition at family level for Betaproteobacteria.

The composition and structure of bacterial communities (Beta diversity) between the various habitats and in the various larvae were evaluated with Bray–Curtis’s dissimilarity matrix and visualized with a nonmetric multidimensional (NMDS) ordination plot (Figs 2D and 2E). Samples did not show any clear clustering pattern either on type (non-productive, semi-productive, or productive) or on description (larval stage 1, stage 2, stage 3 or stage 4) (Fig 1E). A PERMANOVA analysis showed significant differences between bacterial profiles of the samples (Description: R^2 ^= 0.246, P = 0.014 and Type: R^2 ^= 0.072, P = 0.324). The ANOSIM test showed that there were statistical differences between mosquito larvae stages (R = 0.28, P = 0.005) but not for different habitat types (R = -0.08, P = 0.853).

### Comparison of bacterial profiles and diversity between sites.

To determine the similarities and differences between the two sampling sites, samples were compared in different locations (Ada Foah or Dodowa), and the specimen types (water or larvae). This generated 4 sample types: Ada Productive Water, Ada Productive Larvae, Dodowa Productive Water and Dodowa Productive Larvae samples. Using a 0.5% cut off relative abundance, a total of 15 taxa were identified at the class level across all habitats. The most abundant class ASV across all the productive habitats was the *Betaproteobacteria* (Fig 2A), with an average of 51.2%. The common ASVs classes in all habitat groups were *Bacilli, Alphaproteobacteria, Betaproteobacteria, Cytophagia, Flavobacteria, Gammaproteobacteria* and *Mammalia* (Fig 2A). The *Betaproteobacteria* (62.2% and 54.8%) and *Cytophagia* (8.89% and 12.5%) were the dominant taxa in the water samples from the two sites (Ada Foah and Dodowa), while the *Betaproteobacteria* (59.9% and 35.9%) and *Gammaprobacteria* (17.9% and 25.4%) were the dominant classes in the larvae samples (Fig 2A). *Actinomycetia* were only found exclusively in the water samples from both sites, while *Oligoflexia, Opitutae,* and *Saprospiria* were only found exclusively in water samples collected from Ada Foah (Fig 2A). Collectively, the bacterial communities were much similar between water samples and larval samples.

**Fig 3:**
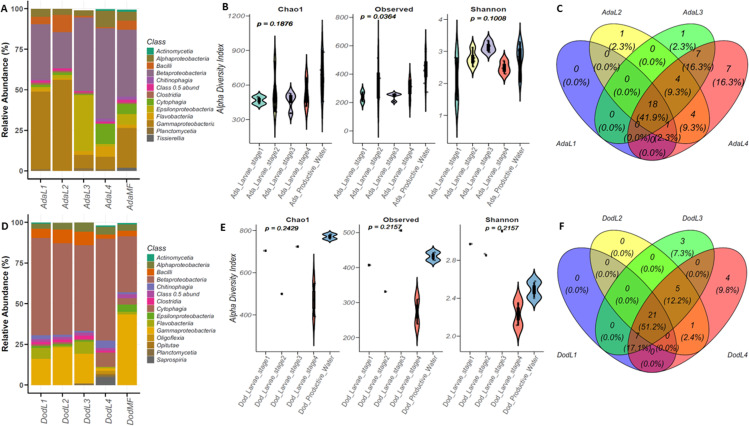
Bacterial profiles and diversity across habitat types and larval stages (L1, L2, L3 and L4) in Ada Foah and Dodowa. Bacterial community composition in An. gambiae larval stages collected from (A) Ada Foah site classified at class level and (**B**) Dodowa site classified at class level. Mean alpha diversity metrics calculated within the various sample groups by (i) Observed species richness ii) Chaol (iii) Shannon diversity index in (**C**) Ada Foah site (**D**) Dodowa site. Four-way Venn diagram depicting ASVs numbers that overlap and do not overlap between different larval stages (L1, L2, L3 and L4) in (**E**) Ada Foah (**F**) Dodowa.

The two dominant classes (*Betaproteobacteria* and *Gammaproteobacteria*) for the predominant bacteria were presented by filtering orders with greater than 5% abundance (Figs 3B and 3C). For the *Gammaproteobacteria,* the order *Enterobacterales* was the dominant order, and this occurred across all sample’s groups, and in the *Betaproteobacteria*, the order *Burkholderiales* was the dominant order.

### Comparison of bacterial profiles and diversity between mosquito larval stages

To determine the similarities and differences between the different larval stages, we compared the bacterial abundances, and diversities in the different larvae stages (L1, L2, L3 and L4) from the two study sites. In general, the bacterial taxa identified in larvae stages closely mirrored bacterial taxa in the water from which they were collected at the different sites (Figs 3A and 3B). For the different larval stages in samples from Ada Foah (Fig 3A), a total of 15 taxa were identified at the class level. *Betaproteobacteria* and *Gammaproteobacteria* were the predominant class across all the larvae stages in the habitats. Several bacterial classes were identified in all larval stages, particularly *Cytophagia*, *Bacilli, Flavobacteria,* and *Epsilonproteobacteria*. These bacterial taxa occurred across all larval stages but differed in abundance between larvae stages. For the different larval stages in samples from Dodowa (Fig 3B), a total of 14 taxa were identified at the class level. The *Betaproteobacteria, Epsilonproteobacteria* and *Gammaproteobacteria* were the predominant classes across all the larvae stages except for the L4 stage. In the L4 stage larvae from the Dodowa habitat the predominant bacterial class was the *Episilonproteobacteria*, but *Gammaproteobacteria* were not identified. Bacterial classes such as *Bacilli, Alphaproteobacteria, Flavobacteria,* and *Cytophagia* were also common across different larvae stages in different abundance (Fig 3B). In comparing the larval stages in the different locations, a distinct observation was the presence of *Actinomycetia* in the water samples in both locations but absent in the various larvae stages. The class *Verrucomicrobiae* was present in the L4 stage in Ada Foah samples but absent across all larvae stages in the Dodowa samples. In a similar manner, *Nitrospra* was identified in the L3 stage in Dodowa samples but absent from the Ada Foah samples (Figs 3A and 3B).

The alpha diversity estimates for the larval stages revealed the richness index showed a statistically significant difference (P = 0.0364, S4 Table), with the highest mean index being the larval stage 2 (336) and the lowest being larval stage 1 (240) (Fig 3C, S4 Table). The Chao1 and Shannon indexes were not significant (p = 0.188 and p = 0.101). For Chao1 the pattern was consistent with the Observed index, with the highest mean index in the larval stage 2 (542) and the larval stage 3 being the lowest (465) (Fig 3C [Chao1]). For the Shannon index, the highest mean was larval stage 3 (3.14) and the lowest was larval stage 1 (2.15) (Fig 3D [Shannon]). All alpha diversity estimates for the larval stages from Dodowa were not statistically significant (Observed: P = 0.216, Chao1: P = 0.243, and Shannon: P = 0.215) (Fig 3D). For the Observed diversity, the highest mean index was in larvae stage 3 (506) and the lowest in larval stage 4 (274) (Fig 3D[Observed]). For Chao1, the pattern was consistent with the Observed index, with the highest mean index in the larvae stage 3 (723) and the lowest mean index in larvae stage 4 (471) (Fig 3D [Chao1]). Similarly, the highest mean Shannon index was in larvae stage 3 (3.11), and the lowest was in larval stage 4 (2.23) (Fig 3D [Shannon]).

A four-way Venn diagram was generated to compare the number of ASVs that overlap between the larval stages (Fig 3E and 3F). For larval stages from Ada Foah, there were 18 ASVs (41.9%) overlap across all stages, with the largest similarity occurring between the L3 and L4 stages (29 ASVs (67.5%) (Fig 3E, S5 Table). Except for larvae stage 1, all other stages had distinctly associated ASVs. For both larvae stages 2 and stage 3, the only unique taxa identified were *Thermoleophilia* and *Coriobacteriia* respectively. Larvae stage 4 had the highest number of unique ASVs (7) these were *Blastocatellia Candidatus, Brocadiia Candidatus, Saccharimonia Candidatus, Thermofonsia, Chlorobia, Thermodesulfovibrionia,* and *Vicinamibacteria* (Fig 3E)*.* For larvae from Dodowa, there were 21 ASVs (51.2%) that overlapped across all stages; as in the Dodowa samples, the largest similarity occurred between the L3 and L4 stages (33 ASVs = 80.5%) (Fig 3F). There were no unique ASVs associated with larvae stages 1 and 2. Larvae stage 3 had 3 unique ASVs (7.3%) (*Acidimicrobiia*, *Chlorobia,* and Chrysiogenetes) while larval stage 4 had 4 unique ASVs (9.8%) and these were *Blastocatellia, Candidatus, Saccharimonia, Pisoniviricetes,* and *Thermoleophilia* (Fig 3F, S5 Table)*.* The Four-way venn diagram depicting ASVs that overlap and do not overlap between various larvae in the different sites at the genus and species taxonomic level is presented in S5 Table.

## Discussion

### Abundance of bacterial in productive habitats

Information about bacteria that either enhance or hamper the survival of mosquito larvae has the potential to be useful in different aspects of mosquito vector control. In this study, the bacterial community composition of *Anopheles* mosquitoes breeding waters in Ghana was evaluated using 16S rRNA gene amplicon sequencing. In this study, productive habitats and their larvae from all the sites had similar bacterial composition with *Gammaproteobacteria* and *Betaproteobacteria* dominating. The two dominant classes in the productive habitats are consistent with previous studies by Nilsson *et al*., [28] in Latin America with *Anopheles darlingi* breeding habitats and in Cameroon with *An. coluzzii* and *An. gambiae* breeding waters by Buck *et. al*., [34]. Similarly, Dada *et al*., [7] have reported the dominance of *Gammaproteobacteria*, and *Betaproteobacteria* in domestic water storage containers containing with *Aedes aegypti* in Thailand and Laos. However, in semi-natural *Anopheles gambiae* breeding sites in Kenya, Wang *et al*., [12] reported *Alphaproteobacteria* and *Cyanobacteria* were abundant in the domestic water-storage containers with *Anopheles* and *Aedes,* and in India, Nilsson *et al.*, [27] detected *Alphaproteobacteria* and *Betaproteobacteria* were abundant in potential *Aedes* and *Anopheles* breeding sites. In eastern Puerto Rico, Caragata, *et al*., [35] detected the abundance of *Alphaproteobacteria*, *Deltaproteobacteria*, and *Epsilonproteobacteria* in *Aedes aegypti* associated breeding habitats water, and in the USA, Njoroge *et al*., [36] detected *Gammaproteobacteria* (*Enterobacteriaceae*) and *Epsilonproteobacteria* (*Arcobacter*) in water samples with *Culex* mosquito larvae, suggesting that there are some class-specific differences in bacteria that are associated with different species of mosquitoes positive breeding habitats.

### Prevalence of bacterial in non-productive habitats

The selection of larval breeding habitats is a critical stage in the life history of gravid mosquitoes because it can ultimately influence the fitness of progeny, distribution of larvae, and larval population dynamics [37]. Selection of breeding habitat sites by gravid mosquitoes can be stimulated by the presences of mixture(s) of bacteria [9,10] and odours emitted from the living microorganisms in soil and water in the habitats [11]. In this study, *Epsilonproteobacteria* and *Actinomycetia* were abundant in habitats where mosquito larvae were absent (non-productive). In contrast to this study, Nilsson *et al*., [27] found that in the absence of mosquito larvae, *Acidimicrobiia, Sphingobacteria* and *Bacilli* were significantly abundant, and the family *Flavobacterium* with *Rhodobacter* was detected in mosquito-negative waters by Dada *et al.,* [7]. *Epsilonproteobacteria* are currently considered to be exclusively hydrogen-oxidizing bacteria [38] and *Actinobacteria* is a class of bacteria that contains nonfilamentous forms of bacteria that produce a large number of biologically active secondary metabolites [39]. Metabolites from *Actinobacteria* have been shown to have strong larvicidal activity against larvae of *Anopheles, Aedes* [39,40], and *Culex* [41] mosquitoes. The finding of *Epsilonproteobacteria* and *Actinomycetia* being in waters devoid of mosquitoes could suggest that there are potential candidates that could be explored for repelling the mosquitoes from ovisiting in potential breeding habitats. These groups of bacteria, and the environmental conditions they reflect, should be further investigated for vector control purposes.

### Bacterial diversity and abundance

There were slight differences in bacterial richness within the water and larval samples studied at the different sites. The Observed (richness) showed that the microbiota of breeding habitats were richer compared to the larvae they contain (Fig 1B). This suggests that the sampling site environment is a key determinant of the bacterial profiles in mosquitoes’ larvae and habitats. Compared to Dodowa, Ada Foah breeding sites were located in farming communities, and the exposure of the bacterial communities in the habitats to pesticides can have some influence on bacterial richness in the aquatic environments [42], partly due to the pesticides suppressing the bacteria in habitats, thus favouring the growth and enrichment of more bacterial species that can degrade the pesticides in the larvae and enable their survival. The importance of the environmental impact of pesticides in shaping the bacterial communities of juvenile mosquitoes and their environment has been documented by Juma *et al*., [43]. Based on findings from this study, there is the need for future studies to evaluate mosquito larvae microbial responses to exposures to pesticides and their possible implications for mosquito ecology and vector control.

Location-driven variability in bacterial composition may arise in various breeding habitats; however, studies have shown that the presence of specific bacterial groups correlates with the presence of mosquito larvae [7,27,44]. Altogether, our investigation showed different bacterial profiles for the different larval stages; however, a fundamental group of bacterial classes predominates at the different stages, with changing abundances. In line with Mosquera *et al.*, [45] study, *Gammaproteobacteri*a and *Betaproteobacteria* were the main ASVs linked to *Anopheles* larvae. However, their variation in proportion during the development of the larvae likely reflects that there might be some selection induced by the alkaline environment in the larval gut due to the presence of the microorganisms [46].

## Conclusion

We present results of the bacterial composition of *Anopheles* larvae and water from potential *Anopheles* larvae productive and non-productive habitats in Ghana. Overall, our findings show that the different habitat types (productive and non-productive) have different bacterial profiles. The Gamma- and Beta- proteobacteria classes that were dominant across the different sample types accounted for as much as 50% of bacterial similarities across the samples . This paper contributes information on the bacterial communities that may directly or indirectly be linked to the absence or presence of mosquitoes. Increasing the understanding of bacterial community composition in breeding sites over time, together with larval densities and mosquito habitat selection, will be important for vector control strategies.

In summary, our data suggest that some bacterial groups are inducing or indicating conditions that are suitable or detrimental for *Anopheles* larvae. This study provides evidence of the presence of potential bacterial microbiota that can be used to develop novel approaches for mosquito control in the future.

## Supporting information

S1 Table Sample sequenced and distribution across different study sites and habitats. (DOCX)

S2 Table Post hoc Dunn test identified statistically significant differences in Observed diversity index between *An. gambiae* larvae and water samples. (DOCX)

S3 Table Unique taxon at class level identified for various habitats. (DOCX)

S4 Table Post hoc Dunn test identified statistically significant differences in Observed diversity index between *An. gambiae* larvae stages collected from the Ada Foah location. (DOCX)

S5 Table Unique taxon at class level identified for larvae stages from Ada Foah and Dodowa location. (DOCX)

S1 Fig Bacterial (Phylum level) community composition of *An. gambiae*, larvae and their habitat sites. (TIF)

S2 Fig Bacterial (Class level) community composition of *An. gambiae*, larvae and their habitat sites. (TIF)

## References

[1] Mutuku FM, King CH, Mungai P, Mbogo C, Mwangangi J, Muchiri EM, et al. Impact of insecticide-treated bed nets on malaria transmission indices on the south coast of Kenya. Malar J. 2011;10:356. doi: 10.1186/1475-2875-10-356 22165904 PMC3322380

[2] Cohen JM, Smith DL, Cotter C, Ward A, Yamey G, Sabot OJ, et al. Malaria resurgence: a systematic review and assessment of its causes. Malar J. 2012;11:122. doi: 10.1186/1475-2875-11-122 22531245 PMC3458906

[3] Ricci I, Damiani C, Capone A, DeFreece C, Rossi P, Favia G. Mosquito/microbiota interactions: from complex relationships to biotechnological perspectives. Curr Opin Microbiol. 2012;15(3):278–84. doi: 10.1016/j.mib.2012.03.004 22465193

[4] Guégan M, Zouache K, Démichel C, Minard G, Tran Van V, Potier P, et al. The mosquito holobiont: fresh insight into mosquito-microbiota interactions. Microbiome. 2018;6(1):49. doi: 10.1186/s40168-018-0435-2 29554951 PMC5859429

[5] Gao H, Cui C, Wang L, Jacobs-Lorena M, Wang S. Mosquito microbiota and implications for disease control. Trends Parasitol. 2020;36(2):98–111. doi: 10.1016/j.pt.2019.12.001 31866183 PMC9827750

[6] Dickson LB, Jiolle D, Minard G, Moltini-Conclois I, Volant S, Ghozlane A, et al. Carryover effects of larval exposure to different environmental bacteria drive adult trait variation in a mosquito vector. Sci Adv. 2017;3(8):e1700585. doi: 10.1126/sciadv.1700585 28835919 PMC5559213

[7] Dada N, Jumas-Bilak E, Manguin S, Seidu R, Stenström TA, Overgaard HJ. Comparative assessment of the bacterial communities associated with Aedes aegypti larvae and water from domestic water storage containers. Parasit Vectors. 2014;7:391. doi: 10.1186/1756-3305-7-391 25151134 PMC4156648

[8] Coon KL, Brown MR, Strand MR. Mosquitoes host communities of bacteria that are essential for development but vary greatly between local habitats. Mol Ecol. 2016;25(22):5806–26. doi: 10.1111/mec.13877 27718295 PMC5118126

[9] Huang J, Miller JR, Chen SC, Vulule JM, Walker ED. Anopheles gambiae (Diptera: Culicidae) oviposition in response to agarose media and cultured bacterial volatiles. J Med Entomol. 2006;43(3):498–504. doi: 10.1603/0022-2585(2006)43[498:agdcoi]2.0.co;2 16739407

[10] Ponnusamy L, Xu N, Nojima S, Wesson DM, Schal C, Apperson CS. Identification of bacteria and bacteria-associated chemical cues that mediate oviposition site preferences by Aedes aegypti. Proc Natl Acad Sci U S A. 2008;105(27):9262–7. doi: 10.1073/pnas.0802505105 18607006 PMC2443818

[11] Sumba LA, Guda TO, Deng AL, Hassanali A, Beier JC, Knols BGJ. Mediation of oviposition site selection in the African malaria mosquito *Anopheles gambiae* (Diptera: Culicidae) by semiochemicals of microbial origin. JTI. 2004;24(03). doi: 10.1079/ijt200433

[12] Wang X, Liu T, Wu Y, Zhong D, Zhou G, Su X, et al. Bacterial microbiota assemblage in Aedes albopictus mosquitoes and its impacts on larval development. Mol Ecol. 2018;27(14):2972–85. doi: 10.1111/mec.14732 29845688 PMC6380897

[13] Girard M, Martin E, Vallon L, Raquin V, Bellet C, Rozier Y, et al. Microorganisms associated with mosquito oviposition sites: implications for habitat selection and insect life histories. Microorganisms. 2021;9(8):1589. doi: 10.3390/microorganisms9081589 34442667 PMC8401263

[14] Lindh JM, Kännaste A, Knols BGJ, Faye I, Borg-Karlson AK. Oviposition responses of Anopheles gambiae s.s. (Diptera: Culicidae) and identification of volatiles from bacteria-containing solutions. J Med Entomol. 2008;45(6):1039–49. doi: 10.1603/0022-2585(2008)45[1039:oroags]2.0.co;2 19058627

[15] Rodgers FH, Gendrin M, Wyer CAS, Christophides GK. Microbiota-induced peritrophic matrix regulates midgut homeostasis and prevents systemic infection of malaria vector mosquitoes. PLoS Pathog. 2017;13(5):e1006391. doi: 10.1371/journal.ppat.1006391 28545061 PMC5448818

[16] Dong Y, Manfredini F, Dimopoulos G. Implication of the mosquito midgut microbiota in the defense against malaria parasites. PLoS Pathog. 2009;5(5):e1000423. doi: 10.1371/journal.ppat.1000423 19424427 PMC2673032

[17] Mathilde G, George K. The anopheles mosquito microbiota and their impact on pathogen transmission. In: Sylvie M, editor. Anopheles mosquitoes. Rijeka: IntechOpen. 2013. p. Ch. 17.

[18] Dennison NJ, Jupatanakul N, Dimopoulos G. The mosquito microbiota influences vector competence for human pathogens. Curr Opin Insect Sci. 2014;3:6–13. doi: 10.1016/j.cois.2014.07.004 25584199 PMC4288011

[19] Gaio A de O, Gusmão DS, Santos AV, Berbert-Molina MA, Pimenta PFP, Lemos FJA. Contribution of midgut bacteria to blood digestion and egg production in aedes aegypti (diptera: culicidae) (L.). Parasit Vectors. 2011;4:105. doi: 10.1186/1756-3305-4-105 21672186 PMC3125380

[20] Abdul Gafaru M, Tamal CS, Odikro MA, Noora CL, Quarshie PR, Afari EA, Kenu E. Epidemiology of malaria cases, Sunyani municipality, Ghana, 2020. J Int Epidemiol Public Health. 2024;1(31).

[21] USAID, US President Malaria Initiative. Entomological Monitoring of The PMI IRD Program In Northern Ghana, Annual Report. 2017; Source: https://www.pmi.gov/docs/default-source/default-document-library/implementing-partner-reports/ghana-2017-entomological-monitoring-final-report.pdf.

[22] Dadzie SK, Brenyah R, Appawu MA. Role of species composition in malaria transmission by the Anopheles funestus group (Diptera: Culicidae) in Ghana. J Vector Ecol. 2013;38(1):105–10. doi: 10.1111/j.1948-7134.2013.12015.x 23701614

[23] de Souza D, Kelly-Hope L, Lawson B, Wilson M, Boakye D. Environmental factors associated with the distribution of Anopheles gambiae s.s in Ghana; an important vector of lymphatic filariasis and malaria. PLoS One. 2010;5(3):e9927. doi: 10.1371/journal.pone.0009927 20360950 PMC2847902

[24] Hinne IA, Attah SK, Mensah BA, Forson AO, Afrane YA. Larval habitat diversity and Anopheles mosquito species distribution in different ecological zones in Ghana. Parasit Vectors. 2021;14(1):193. doi: 10.1186/s13071-021-04701-w 33827667 PMC8025514

[25] Smith TA, Pemberton-Ross P, Penny MA, Chitnis N. Resurgence of malaria infection after mass treatment: a simulation study. Malar J. 2019;18(1):409. doi: 10.1186/s12936-019-3019-0 31805947 PMC6896478

[26] Nkumama IN, O’Meara WP, Osier FHA. Changes in malaria epidemiology in africa and new challenges for elimination. Trends Parasitol. 2017;33(2):128–40. doi: 10.1016/j.pt.2016.11.006 27939610 PMC6995363

[27] Nilsson LKJ, Sharma A, Bhatnagar RK, Bertilsson S, Terenius O. Presence of Aedes and Anopheles mosquito larvae is correlated to bacteria found in domestic water-storage containers. FEMS Microbiol Ecol. 2018;94(6):10.1093/femsec/fiy058. doi: 10.1093/femsec/fiy058 29617987

[28] Nilsson LKJ, de Oliveira MR, Marinotti O, Rocha EM, Håkansson S, Tadei WP, et al. Characterization of bacterial communities in breeding waters of anopheles darlingi in manaus in the amazon basin malaria-endemic area. Microb Ecol. 2019;78(4):781–91. doi: 10.1007/s00248-019-01369-9 30989355 PMC6842340

[29] Onchuru TO, Ajamma YU, Burugu M, Kaltenpoth M, Masiga D, Villinger J. Chemical parameters and bacterial communities associated with larval habitats of *Anopheles*, *Culex* and *Aedes* mosquitoes (Diptera: Culicidae) in western Kenya. Int J Trop Insect Sci. 2016;36(03):146–60. doi: 10.1017/s1742758416000096

[30] Gimonneau G, Tchioffo MT, Abate L, Boissière A, Awono-Ambéné PH, Nsango SE, et al. Composition of Anopheles coluzzii and Anopheles gambiae microbiota from larval to adult stages. Infect Genet Evol. 2014;28:715–24. doi: 10.1016/j.meegid.2014.09.029 25283802

[31] Forson AO, Hinne IA, Sraku IK, Afrane YA. Larval habitat stability and productivity in two sites in Southern Ghana. Malar J. 2023;22(1):74. doi: 10.1186/s12936-023-04498-2 36864430 PMC9983185

[32] Srinivasan R, Karaoz U, Volegova M, MacKichan J, Kato-Maeda M, Miller S, et al. Use of 16S rRNA gene for identification of a broad range of clinically relevant bacterial pathogens. PLoS One. 2015;10(2):e0117617. doi: 10.1371/journal.pone.0117617 25658760 PMC4319838

[33] Miller CS, Handley KM, Wrighton KC, Frischkorn KR, Thomas BC, Banfield JF. Short-read assembly of full-length 16s amplicons reveals bacterial diversity in subsurface sediments. PLoS ONE. 2013;8(2):e56018. doi: 10.1371/journal.pone.0056018 PMC3566076 23405248

[34] Buck M, Nilsson LKJ, Brunius C, Dabiré RK, Hopkins R, Terenius O. Bacterial associations reveal spatial population dynamics in Anopheles gambiae mosquitoes. Sci Rep. 2016;6:22806. doi: 10.1038/srep22806 26960555 PMC4785398

[35] Caragata EP, Otero LM, Tikhe CV, Barrera R, Dimopoulos G. Microbial diversity of adult aedes aegypti and water collected from different mosquito aquatic habitats in puerto rico. Microb Ecol. 2022;83(1):182–201. doi: 10.1007/s00248-021-01743-6 33860847 PMC11328149

[36] Njoroge TM, Berenbaum MR, Stone CM, Kim CH, Dunlap C, Muturi EJ. Culex pipiens and Culex restuans larval interactions shape the bacterial communities in container aquatic habitats. FEMS Microbes. 2024;5:xtae002. doi: 10.1093/femsmc/xtae002 38450098 PMC10917442

[37] Bentley MD, Day JF. Chemical ecology and behavioral aspects of mosquito oviposition. Annu Rev Entomol. 1989;34:401–21. doi: 10.1146/annurev.en.34.010189.002153 2564759

[38] Kruse S, Goris T, Westermann M, Adrian L, Diekert G. Hydrogen production by Sulfurospirillum species enables syntrophic interactions of Epsilonproteobacteria. Nat Commun. 2018;9(1):4872. doi: 10.1038/s41467-018-07342-3 30451902 PMC6242987

[39] Pathalam G, Savarimuthu I. Metabolites from actinobacteria for mosquito control. In: Wael N, editor. Actinobacteria. Rijeka: IntechOpen. 2022. p. 6.

[40] Balakrishnan S, Santhanam P, Srinivasan M. Larvicidal potency of marine actinobacteria isolated from mangrove environment against Aedes aegypti and Anopheles stephensi. J Parasit Dis. 2017;41(2):387–94. doi: 10.1007/s12639-016-0812-3 28615847 PMC5447589

[41] Karthik L, Gaurav K, Rao KVB, Rajakumar G, Rahuman AA. Larvicidal, repellent, and ovicidal activity of marine actinobacteria extracts against Culex tritaeniorhynchus and Culex gelidus. Parasitol Res. 2011;108(6):1447–55. doi: 10.1007/s00436-010-2193-3 21153420

[42] Muturi EJ, Donthu RK, Fields CJ, Moise IK, Kim CH. Effect of pesticides on microbial communities in container aquatic habitats. Sci Rep. 2017;7:44565. doi: 10.1038/srep44565 28300212 PMC5353589

[43] Juma EO, Allan BF, Kim CH, Stone C, Dunlap C, Muturi EJ. Effect of life stage and pesticide exposure on the gut microbiota of Aedes albopictus and Culex pipiens L. Sci Rep. 2020;10(1):9489. doi: 10.1038/s41598-020-66452-5 32528116 PMC7289809

[44] Bascuñán P, Niño-Garcia JP, Galeano-Castañeda Y, Serre D, Correa MM. Factors shaping the gut bacterial community assembly in two main Colombian malaria vectors. Microbiome. 2018;6(1):148. doi: 10.1186/s40168-018-0528-y 30149801 PMC6112144

[45] Mosquera KD, Nilsson LKJ, de Oliveira MR, Rocha EM, Marinotti O, Håkansson S, et al. Comparative assessment of the bacterial communities associated with Anopheles darlingi immature stages and their breeding sites in the Brazilian Amazon. Parasit Vectors. 2023;16(1):156. doi: 10.1186/s13071-023-05749-6 37127597 PMC10150499

[46] Jhuma S, Sibnarayan D. Select bacterial species establish and maintain functionally essential pH gradient profile within the mosquito larval gut. bioRxiv. 2024. doi: 10.1101/2024.02.01.578530

