# Supplementary material for: Assessment of the Bacterial communities associated with Anopheles gambiae larval habitats in Southern Ghana
Source: PLoS One. 2025 May 27;20(5):e0323464. doi: 10.1371/journal.pone.0323464 (PMC12111414; doi:10.1371/journal.pone.0323464)
Supplement: S1 Table — (DOCX) [file pone.0323464.s001.docx]

**S1 Table.** Sample sequenced and distribution across different study sites and habitats

| ***Location*** | **Larvae stage** | **L1** | **L2** | **L3** | **L4** | **Water samples** | **Total** |
| --- | --- | --- | --- | --- | --- | --- | --- |
|  | **Habitat description** |  |  |  |  |  |  |
| **Ada Foah** | Non-productive |  |  |  |  | 4 | **4** |
|  | Semi-productive | 1 | 2 | 2 | 1 | 1 | **7** |
|  | Productive | 1 | 2 | 2 | 2 | 3 | **10** |
| **Dodowa** | Non-productive |  |  |  |  | 1 | **1** |
|  | Productive | 1 | 1 | 1 | 1 | 2 | **6** |
| **Total** | | **3** | **5** | **5** | **4** | **11** | **28** |
