# Supplementary material for: Assessment of the Bacterial communities associated with Anopheles gambiae larval habitats in Southern Ghana
Source: PLoS One. 2025 May 27;20(5):e0323464. doi: 10.1371/journal.pone.0323464 (PMC12111414; doi:10.1371/journal.pone.0323464)
Supplement: S2 Table — (DOCX) [file pone.0323464.s002.docx]

**S2 Table.** Post hoc Dunn test identified statistically significant differences in Observed diversity index between *An. gambiae* larvae and water samples.

| **Comparison** | **Z** | **P.unadj** | **P.adj** |
| --- | --- | --- | --- |
| *** NonProductive_Water - Productive_Larvae** | **1.957** | **0.050** | **0.504** |
| NonProductive_Water - Productive_Water | 0.036 | 0.971 | 1.000 |
| *** Productive_Larvae - Productive_Water** | **-2.077** | **0.038** | **0.378** |
| *** NonProductive_Water - SemiProductive_Larvae** | **2.150** | **0.032** | **0.315** |
| Productive_Larvae - SemiProductive_Larvae | 0.517 | 0.605 | 1.000 |
| *** Productive_Water - SemiProductive_Larvae** | **2.252** | **0.024** | **0.243** |
| NonProductive_Water - SemiProductive_Water | 0.109 | 0.913 | 1.000 |
| Productive_Larvae - SemiProductive_Water | -0.969 | 0.333 | 1.000 |
| Productive_Water - SemiProductive_Water | 0.089 | 0.929 | 1.000 |
| SemiProductive_Larvae - SemiProductive_Water | -1.173 | 0.241 | 1.000 |

(* = P < 0.05)
