# Supplementary material for: Assessment of the Bacterial communities associated with Anopheles gambiae larval habitats in Southern Ghana
Source: PLoS One. 2025 May 27;20(5):e0323464. doi: 10.1371/journal.pone.0323464 (PMC12111414; doi:10.1371/journal.pone.0323464)
Supplement: S3 Table — (DOCX) [file pone.0323464.s003.docx]

**S3 Table.** Unique taxon at class level identified for various habitats.

| ***PRO Water*** | ***PRO Larvae*** | ***SemiPRO Water*** | ***SemiPRO Larvae*** | ***Non-PRO water*** |
| --- | --- | --- | --- | --- |
| *Blastocatellia* | *Chrysiogenetes* | *Holophagae* |  |  |
| *Candidatus Brocadiia* | *Coriobacteriia* |  |  |  |
| *Candidatus Saccharimonia* | *Endomicrobia* |  |  |  |
| *Candidatus Thermofonsia* |  |  |  |  |
| *Chthonomonadetes* |  |  |  |  |
| *Gloeobacteria* |  |  |  |  |
| *Thermodesulfovibrionia* |  |  |  |  |
