# Supplementary material for: Assessment of the Bacterial communities associated with Anopheles gambiae larval habitats in Southern Ghana
Source: PLoS One. 2025 May 27;20(5):e0323464. doi: 10.1371/journal.pone.0323464 (PMC12111414; doi:10.1371/journal.pone.0323464)
Supplement: S4 Table — (DOCX) [file pone.0323464.s004.docx]

**S4 Table.** Post hoc Dunn test identified statistically significant differences in Observed diversity index between *An. gambiae* larvae stages collected from the Ada Foah location.

| **Comparison** | **Z** | **P.unadj** | **P.adj** |
| --- | --- | --- | --- |
| Ada_Larvae_stage1 - Ada_Larvae_stage2 | -0.931 | 0.352 | 1.000 |
| Ada_Larvae_stage1 - Ada_Larvae_stage3 | -0.023 | 0.981 | 1.000 |
| Ada_Larvae_stage2 - Ada_Larvae_stage3 | 1.112 | 0.266 | 1.000 |
| Ada_Larvae_stage1 - Ada_Larvae_stage4 | -0.618 | 0.536 | 1.000 |
| Ada_Larvae_stage2 - Ada_Larvae_stage4 | 0.317 | 0.751 | 1.000 |
| Ada_Larvae_stage3 - Ada_Larvae_stage4 | -0.713 | 0.476 | 1.000 |
| *** Ada_Larvae_stage1 - Ada_Productive_Water** | **-2.155** | **0.031** | **0.312** |
| Ada_Larvae_stage2 - Ada_Productive_Water | -1.465 | 0.143 | 1.000 |
| *** Ada_Larvae_stage3 - Ada_Productive_Water** | **-2.749** | **0.006** | **0.060** |
| Ada_Larvae_stage4 - Ada_Productive_Water | -1.682 | 0.092 | 0.925 |

(* = P < 0.05).
