# Supplementary material for: Assessment of the Bacterial communities associated with Anopheles gambiae larval habitats in Southern Ghana
Source: PLoS One. 2025 May 27;20(5):e0323464. doi: 10.1371/journal.pone.0323464 (PMC12111414; doi:10.1371/journal.pone.0323464)
Supplement: S5 Table — (DOCX) [file pone.0323464.s005.docx]

**S5 Table. Unique taxon at class level identified for larvae stages from Ada Foah and Dodowa location**

|  | **Ada Foah site** | | |
| --- | --- | --- | --- |
| ***AdaL1*** | ***AdaL2*** | ***AdaL3*** | ***AdaL4*** |
|  | *Thermoleophilia* | *Coriobacteriia* | *Blastocatellia* |
|  |  |  | *Candidatus Brocadiia* |
|  |  |  | *Candidatus Saccharimonia* |
|  |  |  | *Candidatus Thermofonsia* |
|  |  |  | *Chlorobia* |
|  |  |  | *Thermodesulfovibrionia* |
|  |  |  | *Vicinamibacteria* |
|  | **Dodowa site** | | |
| ***DodL1*** | ***DodL2*** | ***DodL3*** | ***DodL4*** |
|  |  | *Acidimicrobiia* | *Blastocatellia* |
|  |  | *Chlorobia* | *Candidatus Saccharimonia* |
|  |  | *Chrysiogenetes* | *Pisoniviricetes* |
|  |  |  | *Thermoleophilia* |
