# Supplementary figures and images for: Assessment of the Bacterial communities associated with Anopheles gambiae larval habitats in Southern Ghana
Source: PLoS One. 2025 May 27;20(5):e0323464. doi: 10.1371/journal.pone.0323464 (PMC12111414; doi:10.1371/journal.pone.0323464)

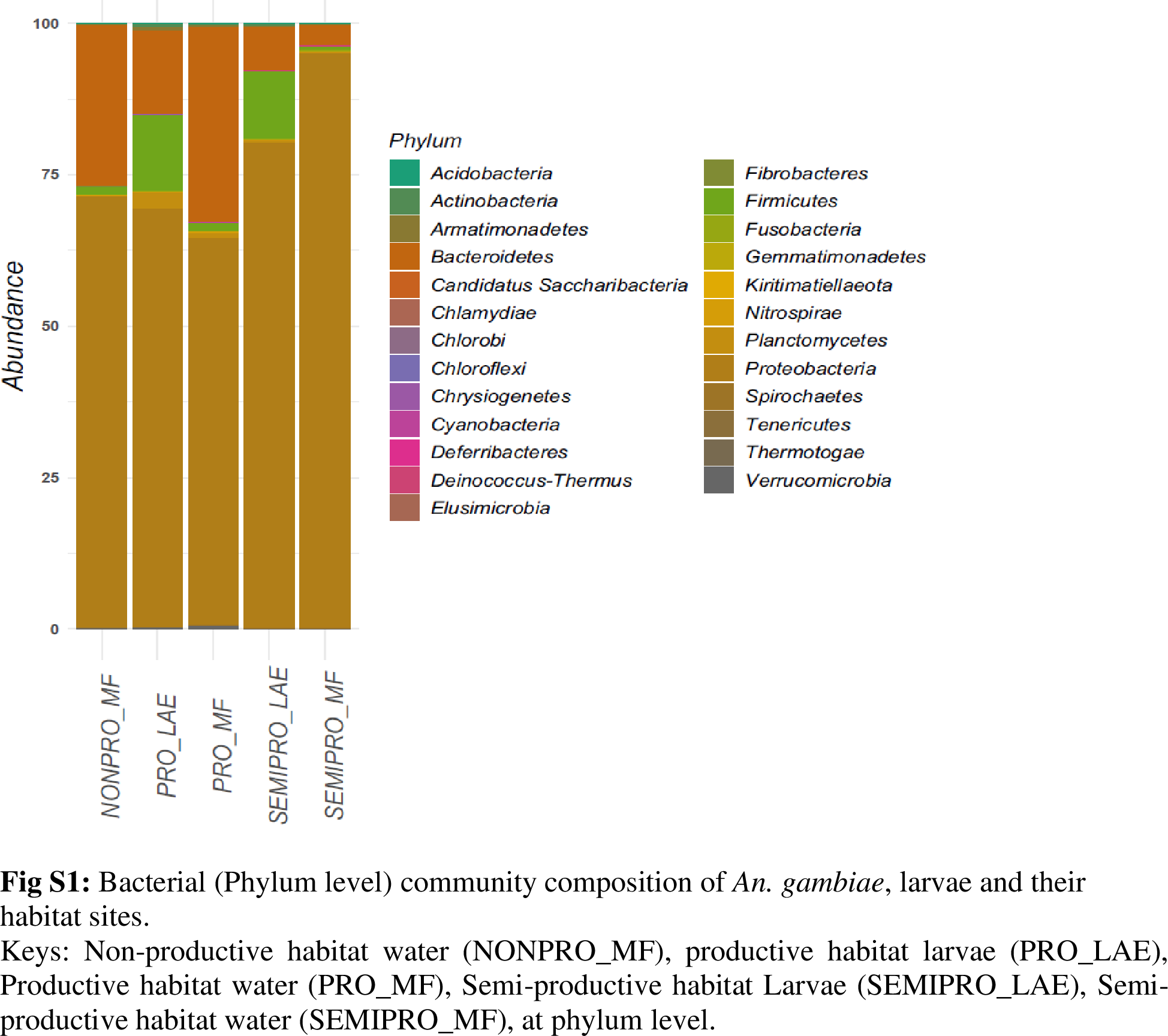

Supplement: S1 Fig — (TIF) [file pone.0323464.s006.tif]

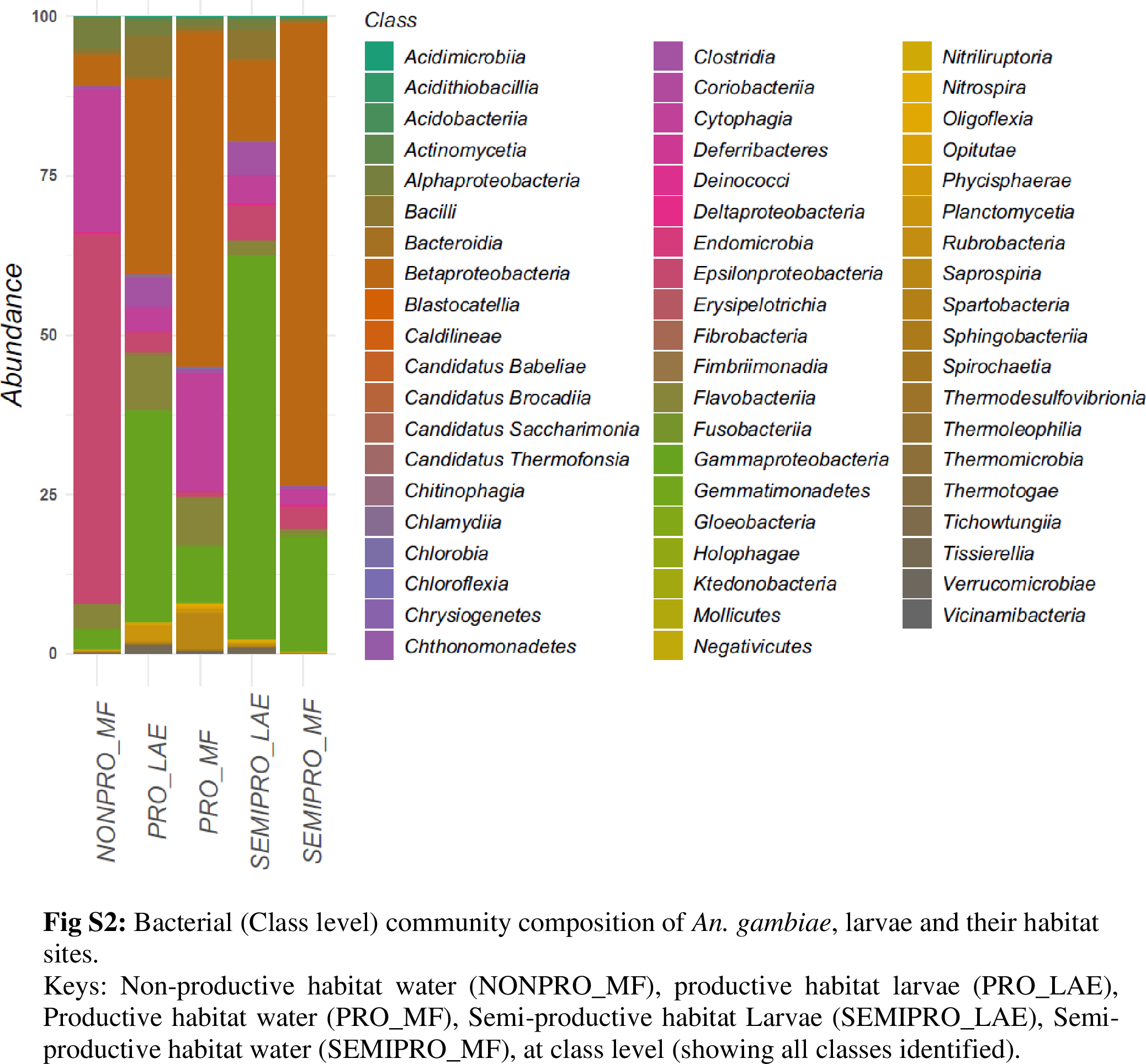

Supplement: S2 Fig — (TIF) [file pone.0323464.s007.tif]
